# Reduction and recovery of keystone predation pressure after disease‐related mass mortality

**DOI:** 10.1002/ece3.3953

**Published:** 2018-03-23

**Authors:** Monica M. Moritsch, Peter T. Raimondi

**Affiliations:** ^1^ Department of Ecology and Evolutionary Biology University of California Santa Cruz Long Marine Laboratory Santa Cruz CA USA

**Keywords:** growth, keystone predator, *Pisaster ochraceus*, recruitment, sea star wasting disease, sea star wasting syndrome

## Abstract

Disturbances such as disease can reshape communities through interruption of ecological interactions. Changes to population demographics alter how effectively a species performs its ecological role. While a population may recover in density, this may not translate to recovery of ecological function. In 2013, a sea star wasting syndrome outbreak caused mass mortality of the keystone predator *Pisaster ochraceus* on the North American Pacific coast. We analyzed sea star counts, biomass, size distributions, and recruitment from long‐term intertidal monitoring sites from San Diego to Alaska to assess regional trends in sea star recovery following the outbreak. Recruitment, an indicator of population recovery, has been spatially patchy and varied within and among regions of the coast. Despite sea star counts approaching predisease numbers, sea star biomass, a measure of predation potential on the mussel *Mytilus californianus*, has remained low. This indicates that post‐outbreak populations have not regained their full predation pressure. The regional variability in percent of recovering sites suggested differences in factors promoting sea star recovery between regions but did not show consistent patterns in postoutbreak recruitment on a coast‐wide scale. These results shape predictions of where changes in community composition are likely to occur in years following the disease outbreak and provide insight into how populations of keystone species resume their ecological roles following mortality‐inducing disturbances.

## 1 INTRODUCTION

Disturbance and mass mortalities can reshape the ability of affected populations to maintain their role within an ecosystem. Disturbances that remove individuals of species with large relative contributions to ecological functions often result in greater changes to community composition (Estes, Smith, & Palmisano, 1978; Lessios, 1988; Paine, 1966). For example, size‐selective fishing of larger individuals from populations of predatory spiny lobster *Jasus edwardsii* and California sheephead *Semicossyphus pulcher* disproportionately decreases those populations’ ability to control urchin grazing on kelp biomass, increasing the chance of overgrazing and transition of kelp forest to urchin barren (Hamilton & Caselle, 2015; Ling, Johnson, Frusher, & Ridgway, 2009). Demographic attributes such as size‐dependent predation and ontogenetically influenced diet preferences can moderate recovery of ecological function. Younger, smaller individuals typically do not consume the same biomass of prey as full‐grown adults (Brodeur, 1991; Feder, 1956). This means that even when a population recovers to the same predisturbance number of individuals, it will not entirely resume its ecological role until more individuals reach adult size and restore predisturbance size structure (Bellwood, Hoey, & Hughes, 2012; Hamilton & Caselle, 2015). This is particularly true for species functioning as keystone predators, which play a large role in maintaining community composition relative to their abundance (Paine, 1966).

Diseases may act as natural disturbances that moderate the strength of affected populations’ ecological interactions (Selakovic, de Ruiter, & Heesterbeek, 2014). Infected hosts can alter their prey consumption of a given species or become highly vulnerable prey for another species. In intertidal habitats, the marine snail *Littorina littorea* decreases its algal consumption when infected by trematodes, subsequently producing changes in intertidal macroalgal community composition (Wood et al., 2007). Behavioral shifts in infected terrestrial insects cause them to enter streams and encounter new predators (Ponton et al., 2011; Sato et al., 2012). In addition to food‐web effects, diseases often cause age‐specific mortality of the host, altering population demography, which in turn impacts the ecological function of the population (Groner et al., 2014). While dynamic cycles of infection and recovery in disease outbreaks are a common mechanism constraining host abundances, epidemics have the potential to cause mass mortalities in host populations beyond the fluctuations experienced in regular cycles because infections and deaths occur at much higher rates than recovery. Therefore, epidemics are expected to have greater ecological consequences (Anderson & May, 1991; Hughes, Deegan, & Wyda, 2002; Leighton, Boom, Bouland, Hartwick, & Smith, 1991; Lessios, 1988; Rockwood, 2006).

As global climate change increases temperatures and alters physical conditions within marine habitats, epizootic diseases and their associated mass mortalities are projected to increase in the coming decades (Baker‐Austin et al., 2012; Burge et al., 2014; Harvell et al., 1999). Intensifying anthropogenic pressures on marine habitats will further contribute to stressful conditions that compromise host immune system activities (Harvell et al., 1999; Mydlarz, Jones, & Harvell, 2006). Altered species interaction strengths stemming from change in abundance or shifts in demographics can have cascading effects throughout the community, especially when the affected host plays a role of major ecological importance such as a keystone, dominant, or foundational species (Hughes et al., 2002; Menge, 1995; Smith, Behrens, & Sax, 2009). As such, understanding the recovery dynamics of infected species is critical to predicting potential changes to the broader community.

**Figure 1:**
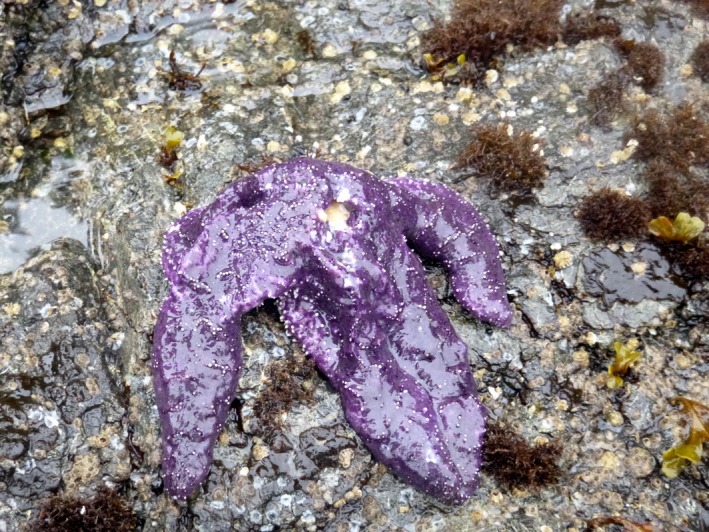
*Pisaster ochraceus* with sea star wasting syndrome symptoms. The body can appear deflated. White lesions appear on the body wall, followed by tissue decay, arm loss, and death

Beginning in summer 2013 and continuing through 2014, an epidemic of sea star wasting syndrome (SSWS; Figure 1) caused major declines in multiple species of echinoderms on the Pacific coast of North America (Hewson et al., 2014; MARINe 2013). The high disease prevalence of the epidemic subsided after 2014, but SSWS was still present in sea star populations at lower prevalence (<20% of sea stars with symptoms) as of 2016 (Figure S3). In rocky intertidal habitats, the ochre star *Pisaster ochraceus* (Brandt) was one of the most dramatically reduced species. Some sites experienced nearly 100% mortality, but considerable spatial variability in mortality patterns was present over larger spatial scales of tens and hundreds of kilometers (Eisenlord et al., 2016; Menge et al., 2016). Levels of recruitment two orders of magnitude higher than average followed in the 1–2 years after mass mortality, shifting *P. ochraceus* size structure toward smaller individuals (Menge et al., 2016).

*Pisaster ochraceus* is recognized as a keystone predator in intertidal habitats because of its ability to reduce the abundance and constrain spatial distributions of the California mussel *Mytilus californianus,* a competitive dominant species (Paine, 1966, 1974). Larger sea stars have the physical strength to consume larger mussels and eat more mussel meat than smaller individuals (Feder, 1956; McClintock & Robnett, 1986; Robles, Desharnais, Garza, Donahue, & Martinez, 2009). While a superabundance of newly settled sea stars exerts substantial predation pressure on mussel recruits (Menge et al., 2016), reduced abundance of adults and demographic shifts to smaller *P. ochraceus* reduce overall predation pressure on *M. californianus* (Menge & Menge, 1974; Robles et al., 2009). Experimental sea star removals in many locations on the Pacific coast have demonstrated that when *P. ochraceus* abundances are severely reduced for a sufficiently long duration, *M. californianus* increase in percent cover (Paine, 1966, 1974; Robles et al., 2009). This expansion of the mussel bed decreases the number of other species on the primary substrate, while it creates more habitat for infaunal and epibiont species (Lafferty & Suchanek, 2016; Suchanek, 1986). Conversely, experimental additions of *P. ochraceus* result in the elimination of larger mussels, a reduction in overall mussel cover, and greater cover of other prey species over time (Robles et al., 2009).

Speed of recovery for sea star populations depends on the magnitude of recruitment and postsettlement mortality, which influences the number of new individuals available to replace those that died (Lessios, 1988; Miner, Altstatt, Raimondi, & Minchinton, 2006). However, assessment of whether a population has returned to historic levels of predation pressure must also incorporate growth rates of sea stars because predation pressure is more related to collective predator biomass than predator abundance. *P. ochraceus* requires three to five years to reach terminal adult size given abundant food availability (Feder, 1970; Pilkerton et al., 2016). As population recovery of *P. ochraceus* progresses, overall predation pressure will show a lagged response as individuals gradually increase in biomass.

Here, we focus specifically on geographic differences in the recovery process, as this improves our understanding of where we may expect community changes and where to direct future intertidal monitoring efforts. We assessed spatial patterns in which populations have begun recovery based on recruitment of *P. ochraceus*. We characterized the spatiotemporal trends in recovery of predation pressure on mussel populations using abundance, biomass, and size structure through comparisons of post‐SSWS populations to long‐term observations preceding the wasting event. We discuss the importance of recruitment and postsettlement mortality as potential contributors to the differential recovery patterns that we observed.

## 2 MATERIALS AND METHODS

We characterize the recovery process using multiple metrics. Recruitment, indicated by sea stars arriving that are too young to have experienced the outbreak, is a marker of postdisease reproduction. While recruitment pulses occurred in some locations during the peak of the outbreak, it is possible that large numbers of those recruits died before reaching maturity due to juveniles’ high SSWS mortality. Those that survived would contribute to recovery, but ultimately true recovery and persistence of the population require successful reproduction and recruitment after the outbreak. From an ecological perspective, recovery requires the return of the population's function within the community in addition to replacement of lost individuals. Sea star biomass serves as a proxy of predation pressure on the mussel bed due to its correlation with prey size and mass of soft tissue consumption (Feder, 1956; Robles et al., 2009). Finally, comparison of pre‐SWSS and post‐SSWS size distributions over several years allows us to evaluate trajectory of ecological recovery by whether it is regaining larger individuals that contribute disproportionately to predation pressure.

### 2.1 Sea star surveys

The Partnership for Interdisciplinary Studies (PISCO) and the Multi‐Agency Rocky Intertidal Network (MARINe) monitor long‐term abundances and sizes of *P. ochraceus* on the Pacific Coast from Southern California to Alaska. To capture trends in populations of intertidal species across a large geographic range, a network of data collection groups coordinates methods for counting and measuring species. The authors were part of the PISCO survey team. The first year of surveys varies between sites, but data are generally available for at least the past decade. Individual sites are surveyed in the same season(s) annually, although because of geographical differences in the timing of suitable tides, not all regions are surveyed in the same season. California sites are generally surveyed in the spring and fall, while sites to the north are surveyed in the summer. Sites containing sea star plots were nonrandomly selected for optimal sea star habitat and logistic feasibility of sampling. Monitoring sites had stable rocky surfaces, low to moderate scour by sand and gravel, and moderate wave protection for safe low tide sampling (MARINe 2008). Our goal was to compare sea star recovery trends in similar habitat types across regions rather than to examine sea star recovery patterns in all habitats where sea stars could survive. To assess geographic trends within and among regions, we designated nine regions as follows (abbreviations in parentheses): Southeast Alaska (AK), Washington Olympic Coast (WA Olympic Coast), Washington Salish Sea (WA Salish Sea), Oregon (OR), North California (CA North), North Central California (CA North Central), Central California (CA Central), California Channel Islands (CA Channel Islands), and South California (CA South). The extent of these regions (Table S1) corresponds to major units in marine management by state governments. These regional designations are consistently used all in PISCO/MARINe surveys.

We used PISCO/MARINe data from spring and summer 2015, 2016, and 2017 (post‐SSWS) and from 1989 to 2012 (pre‐SSWS) for *P. ochraceus* counts and sizes. We did not use fall data from these 3 years because fall 2017 sampling was still in progress at the time of this writing. We excluded data from 2013 and 2014 because the inconsistent timing and monitoring of the disease outbreak between regions during this period prevented definitive designations of sites as preoutbreak or postoutbreak. Count and size data availability for 2015–2017 for each site are summarized in Tables S1–S3. These surveys counted visible sea stars within permanently marked nonstandard polygons (plots) of ideal *P. ochraceus* habitat: middle and low intertidal zones, some containing deep crevices, overhangs, or vertically protruding rock. Each site had three sea star survey plots. Surveyors used flashlights to examine crevices and overhangs to improve visibility, and surveyors moved around the plot to inspect all safely accessible places where a sea star could hide. Despite these steps to improve visibility, it is possible that these rock structures obscured detection of some *P. ochraceus*. A plot's rock structure remained consistent over time, so searchable area of a plot was comparable between years. Due to nonstandardized plot areas, sea star counts from these surveys are able to identify trends but are not able to perform direct site‐to‐site comparison in terms of raw abundances or biomass. With a ruler, the surveyor physically measured the radius (center of body to arm tip) of the longest visible arm of all sea stars in the plot. Each arm measurement was rounded to the nearest 10 mm with the exception of sea stars ≤7 mm, which were binned as 5 mm (MARINe 2008). This smallest size category is often difficult to see in visual field surveys and is likely consistently underrepresented in counts across all years. Sea star counts for all years were maintained in the PISCO/MARINe database. All raw data for *Pisaster ochraceus* counts, sizes, and years are publicly available online at http://www.eeb.ucsc.edu/pacificrockyintertidal/contact/index.html.

### 2.2 Estimation of sea star arrival dates and sites in recovery

We considered the arrival of offspring of survivors the first step in the recovery process. Given the severe mortality from SSWS and the low postoutbreak reproduction in some regions, we defined a site as being “in recovery” if juvenile *P. ochraceus* that were offspring from the spawning of survivors of the initial SSWS mass mortality were present. This definition excluded sites that had not experienced recruitment success since the outbreak. To differentiate offspring of survivors from juveniles that were already alive during the outbreak, we estimated the date of arrival of post‐SSWS recruits at each site. Mass mortalities within a region took place over a few weeks to several months since the first regional observation of SSWS. Most population declines occurred by 4–6 months after the first SSWS observations in the region (Menge et al., 2016). We considered sea stars to have made it through the mass mortality period if they were alive 8 months (240 days) after the first observation of SSWS in their respective regions. This includes a two‐month buffer to be conservative in classifying sea stars as survivors. We designated this eight‐month benchmark as the earliest time of reproduction by SSWS survivors. We assumed that recruits from these survivors would arrive after a 70‐day pelagic larval duration period (Strathmann, 1978), setting the earliest defined date of arrival of new recruits at 310 days after the first observation of SSWS in the region.

*Pisaster ochraceus* broadcast spawn and larvae remain in the water column for several weeks (George, 1999; Strathmann, 1978). This definition of the earliest post‐SSWS arrival date would underestimate offspring from disease survivors that spawned before the end of the mass mortality period, making it a conservative estimate of post‐SSWS sea star numbers. Gene flow is generally high along the open coast with little genetic structure (Frontana‐Uribe, de la Rosa‐Vélez, Enríquez‐Paredes, Ladah, & Sanvicente‐Añorve, 2008; Harley, Pankey, Wares, Grosberg, & Wonham, 2006; Stickle, Foltz, Katoh, & Nguyen, 1992). However, it is possible for sea star populations in embayments and sounds to exhibit higher levels of genetic structure (Keever et al., 2009; Sewell & Watson, 1993). While we did not expect high levels of self‐recruitment, the timing of SSWS mass mortalities was generally similar over the distance scales which larvae would typically travel. Because of our conservative designation of what counted as the offspring of survivors, this method of back‐calculation is robust to within‐region variations in the timing of outbreaks.

We used site‐specific growth rate (Table S4) and 2015–2017 sizes of sea stars to back‐calculate arrival date. We assumed growth rate was linear until sea stars reached adult sizes (Pilkerton et al., 2016; Sewell & Watson, 1993), although this is a simplification of field growth rates, which can vary with food availability (Feder, 1970). All sea stars calculated to arrive at the site after the earliest (post‐SSWS) arrival date were considered to have originated after the SSWS outbreak. Sites with individuals arriving post‐SSWS were considered “in recovery”. Sites with 0% of their individuals arriving post‐SSWS were considered “not in recovery” at the time of surveys.

### 2.3 Influence of SSWS on the *Pisaster ochraceus* reproductive cycle

*Pisaster ochraceus* invest in gonadal growth in September through March and usually spawn in April through June under normal conditions (Mauzey, 1966; Pearse & Eernisse, 1982; Sanford & Menge, 2007). This species has a long pelagic larval duration of 5–32 weeks, after which larvae settle in the intertidal and metamorphose into juveniles (George, 1999; Strathmann, 1978). The new recruits are generally detectable by the following spring (Sewell & Watson, 1993). Had the epidemic not occurred, we likely would have observed stronger seasonal constraints in when larvae arrived. However, in Central California, we observed *P. ochraceus* spawning during the mass mortality period in fall 2013 and winter 2014. Spawning activity was prevalent at high enough levels to include the appearance of spawning stars on SSWS identification guides for citizen science data collection efforts during this period (MARINe 2014). With this disruption in seasonality of the reproductive cycle, we did not apply seasonal constraints to the possible arrival times of recruits.

### 2.4 Degrees of recovery of predation pressure

We considered replenishment of abundance and predation pressure the next steps in recovery. We used sea star biomass as a proxy for predation pressure. Any sites that did not have historical size data were excluded from calculation of size distribution or biomass but still used in analysis of counts (Eel Point and West Cove in CA Channel Islands).

We used PISCO/MARINe sea star survey data from 1989 to 2012 to calculate the pre‐SSWS long‐term means for abundance, biomass, and size distribution. We defined a site's degree of ecological recovery in two ways: (1) individuals present; and (2) biomass present in the plots as a proportion of the site's pre‐SSWS mean. We standardized abundance and biomass to the pre‐SSWS mean because nonstandard plot sizes prevented meaningful among‐site comparisons of absolute numbers.

From the measured arm length, we calculated sea star biomass by performing a linear regression between log‐transformed radius (mm) and log‐transformed mass (g) based on data from the reserve at Hopkins Marine Station by Feder (1956) and our own measurements of 58 individuals from the Central California region:

ln(biomass)=2.34723×ln(radius)−5.50262

To calculate a site's relative predation pressure, we summed the biomass of all individuals present in the site's plots in a given year and divided it by the site's pre‐SSWS mean biomass. The relationship between biomass and predation pressure is simplified for our purposes. Under natural densities, larger *P. ochraceus* tend to consume longer mussels, though the relationship between sea star biomass and mussel meat consumed is not thoroughly explored (Robles et al., 2009). The summation of biomass for all individuals at a site also assumes that biomass is the only factor controlling predation pressure and does not account for spatial patterns of predation by large and small individuals. Larger sea stars can eat more meat, but they more frequently stay in pools or in the low intertidal, where they may not eat as many mussels (Fly, Monaco, Pincebourde, & Tullis, 2012; Petes, Mouchka, Milston‐Clements, Momoda, & Menge, 2008). However, large sea stars can move >3 m during high tide and will aggregate on dense patches of mussel recruits, so they still exert some control over the lower limit of the mussel bed (Robles, Sherwood‐Stephens, & Alvarado, 1995). Small sea stars have a more varied diet, but they also consume small mussels that contribute heavily to mussel bed expansion (Feder, 1959; Menge & Menge, 1974). Despite these limitations as a proxy for absolute predation pressure, biomass combined with size distribution can characterize how predation at a site compares to the preoutbreak state.

If sea star populations had a stable size distribution, we would expect that an increase in the proportion of pre‐SSWS count would result in an equal increase in the proportion of pre‐SSWS biomass. However, with shifts in size distributions resulting from the disease outbreak, numerical gains are not necessarily coupled with proportionate gains in biomass. To examine how relative biomass changes with relative counts, we calculated change in proportion of pre‐SSWS mean counts (% pre‐SSWS count_time 2_ − % pre‐SSWS count_time 1_) and proportion of pre‐SSWS mean biomass (% pre‐SSWS biomass_time 2_ − % pre‐SSWS biomass_time 1_) for each site between 2015 and 2016 (*n* = 60) and between 2016 and 2017 (*n* = 33). We also performed this calculation on changes in consecutive years of proportion of count and biomass (*n* = 268) at each site with pre‐SSWS data. We determined the relationship between count change and biomass change using linear regression for each region and each year‐to‐year period separately. If the slope of the regression was less than the slope of the regression for that of pre‐SSWS count and biomass changes, gains in relative count resulted in less gains than expected to relative biomass, and therefore less gains to the recovery of pre‐SSWS predation pressure. We excluded regions that had fewer than four sites sampled in consecutive post‐SSWS years due to low sample size in change of count and biomass (Table S3).

Comparing preoutbreak and postoutbreak size distributions gives an indication of demographic changes. To determine whether size distributions were approaching or diverging from the pre‐SSWS distributions, we compared 2015 (*n* = 49), 2016 (*n* = 44), and 2017 (*n* = 22) size distributions to the long‐term average size distributions at each site using a Kolmogorov–Smirnov test (hereafter “K–S test”). For each site, the K–S tests generated a *D* statistic, a measure of difference between the long‐term focal year distributions. We averaged the *D* statistic for all sites in a region in each year to assess geographic trends in size distributions (number of sites per region are detailed in Table S2). Linear regressions were performed in JMP Pro 13. All other statistics were performed in R 3.2.2.

## 3 RESULTS

### 3.1 Geographic patterns of population recovery

**Figure 2:**
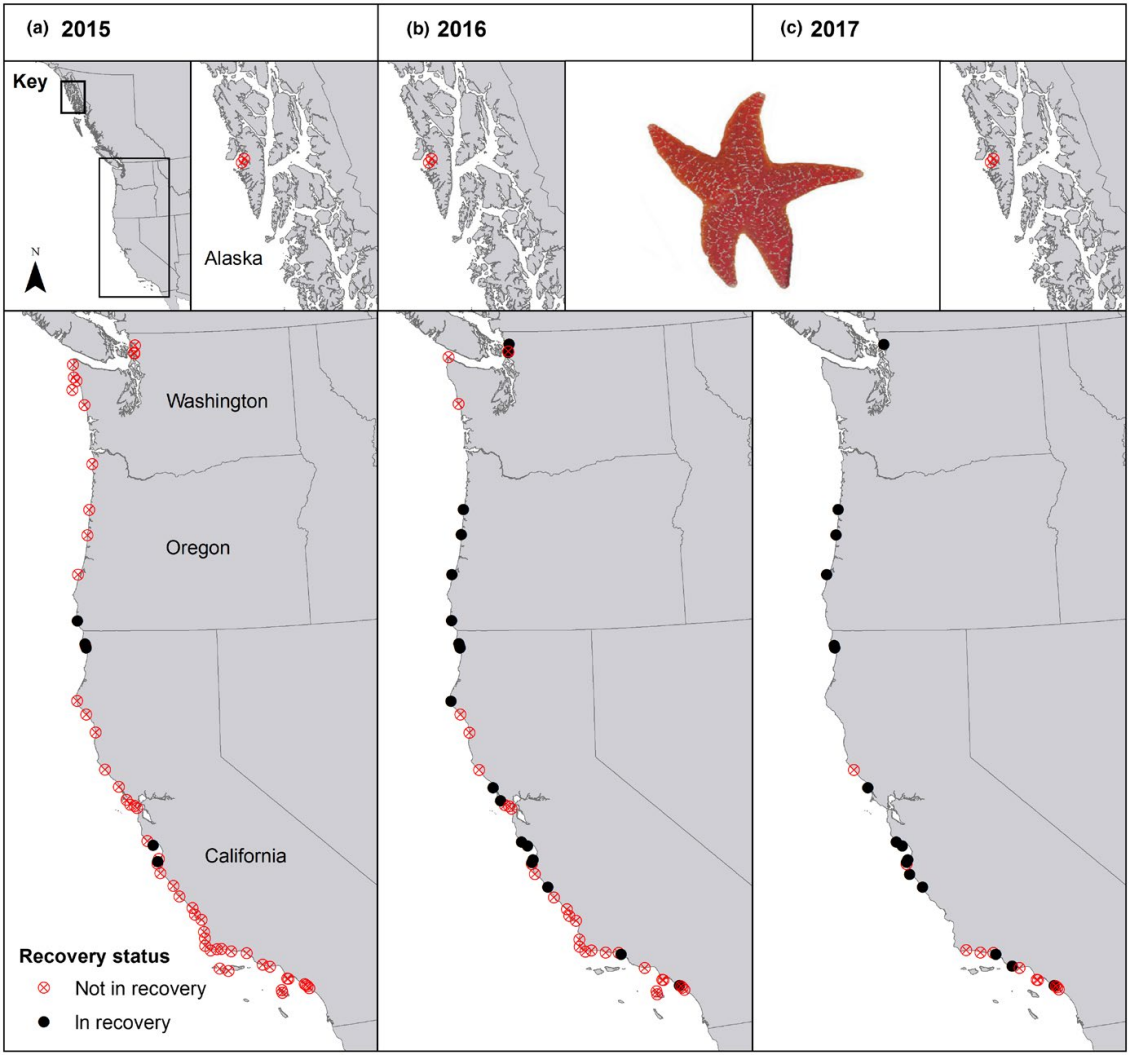
Map of sites that have begun population recovery in (a) 2015, (b) 2016, and (c) 2017. Closed black circles represent sites with post‐SSWS‐born *Pisaster ochraceus* present. Open red circles with an “x” represent sites with no post‐SSWS‐born *P. ochraceus* present

In 2015, few sites coast‐wide were in recovery, defined by the presence of individuals with a post‐SSWS arrival date. Oregon, North California, and Central California were the only regions with recovering sites that year (Figure 2a). We detected post‐SSWS recruits starting in 2016 in Washington Salish Sea, North Central California, and South California. Recovering sites clustered spatially in North and Central California (Figure 2b). In 2017, the percentage of sites in recovery continued to increase in all regions that had recovering sites the previous year (Figure 2c). In contrast, we did not observe post‐SSWS individuals in Southeast Alaska, the Washington Olympic Coast, or the Channel Islands in any of the years sampled (Figure 2a–c). Across all regions, we observed five sites (8%) in recovery in 2015, 19 sites (37%) in 2016, and 16 sites (55%) in our partial year of sampling in 2017.

### 3.2 Geographic patterns of recovery of predation pressure

**Figure 3:**
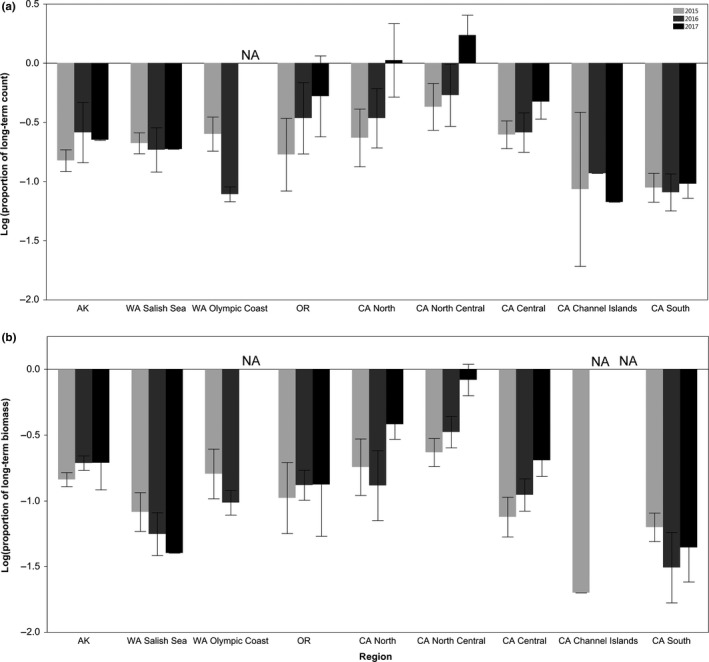
Regional means of (a) log‐transformed counts and (b) log‐transformed biomass of *Pisaster ochraceus* as the proportion of each site's pre‐SSWS mean. Light gray bars represent 2015, medium gray bars represent 2016, and black bars represent 2017. Error bars denote standard error. Sites sampled each year are described in Tables S1 and S3. In 2016 and 2017, all sites sampled in the CA Channel Islands region had either no pre‐SSWS size data or had counts of 0 individuals, preventing calculation of mean biomass

We observed regional differences in the degree of recovery of sea star abundances as well as biomass, our proxy for predation pressure. Oregon, North California, and North Central California had counts approaching or exceeding their pre‐SSWS averages by 2017. From 2015 to 2017, number of individuals increased in these three regions and Central California (Figure 3a). Mean abundance remained approximately the same or declined in Southeast Alaska, Washington Salish Sea, Washington Olympic Coast, the California Channel Islands, and South California over the same time period. These regions had 30% or less of their pre‐SSWS abundances. The Channel Islands and South California had particularly low abundances of 0%–10% of pre‐SSWS averages. The gains in sea star counts were not matched by the same gains in sea star biomass, and by extension, predation pressure was still far below its pre‐SSWS state for most sites in 2017. All regions except for North Central California had below 40% of their respective long‐term biomass in 2015–2017 (Figure 3b). In years where size and biomass data were available, Southern California and the Channel Islands had near‐zero biomass.

**Figure 4:**
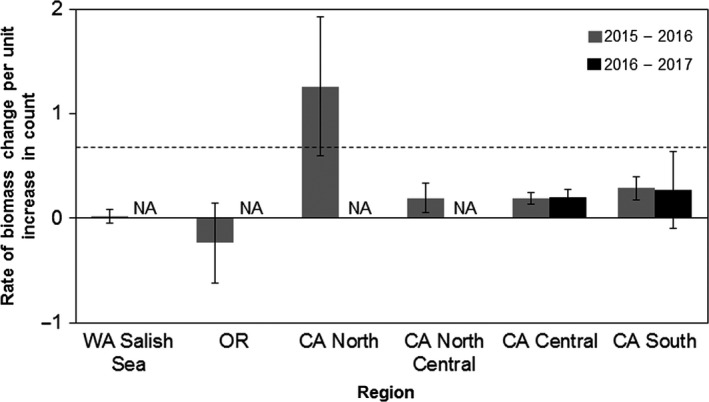
Rate of change in relative biomass per unit increase in relative counts. The height of the bar represents the slope of the linear regression between change in proportion of pre‐SSWS count and change in proportion of pre‐SSWS biomass between two consecutive years. Only regions with enough sites (*n* ≥ 4) in consecutive years are shown. Dashed reference line at *y *=* *0.67 represents the average slope of the linear regression between change in proportion of count and biomass at sites from 1989 to 2012

Gains in relative count were not reflected in equal gains in relative biomass between years. Relative biomass increased at an average of 0.28 (*SE* = 0.19) times the rate that relative count increased between 2015 and 2016 and increased at 0.23 (*SE* = 0.03) times the rate between 2016 and 2017 (Figure 4). This is below the 0.67 (*SE* = 0.02) rate of increase in relative biomass and count that would be expected based on year‐to‐year changes in count and biomass during the pre‐SSWS period. A region could even lose biomass while increasing count if the new individuals were small and mortality was primarily of large individuals. This occurred at some sites in Oregon between 2015 and 2016 and is reflected in the negative relationship between count and biomass for this region (Figure 4).

### 3.3 Shifts in size distribution

**Figure 5:**
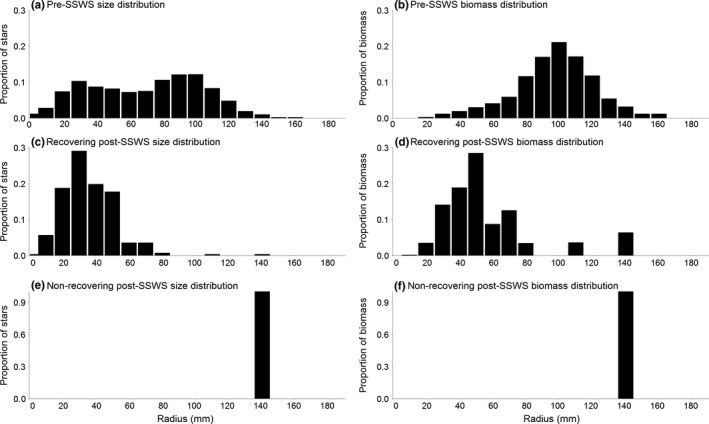
Example size distributions of *Pisaster ochraceus*. (a) Pre‐SSWS sizes are more uniformly distributed throughout the observed size range with increases reflecting strong recruitment years. (b) Pre‐SSWS biomass distributions resemble a normal distribution centered on large sizes. (c) Distribution of sea stars is shifted toward small individuals at recovering sites with high recruitment. (d) Biomass at recovering sites is still concentrated in small to medium individuals. At nonrecovering sites (e), both size and (f) biomass distributions were irregular, shaped by a low number of sea stars (<10) remaining survivors in survey plots. Data for (a, b) from Mill Creek 2017, Monterey County, CA. Data for (c, d) from Mill Creek pre‐SSWS. Data for (e, f) from Coal Oil Point 2017, Santa Barbara County, CA

For sites in recovery (postoutbreak recruits present), smaller individuals comprised a greater proportion of total *P. ochraceus* than large individuals, producing post‐SSWS size and biomass distributions shifted toward smaller body sizes (≤45 mm in radius; Figure 5c,d), whereas in pre‐SSWS distributions, medium and large individuals (≥75 mm) represented the greatest segment of a site's sea stars (Figure 5a,b). Pre‐SSWS populations had multiple normal distributions of individuals reflecting each past recruitment event. Pre‐SSWS biomass resembled a normal distribution across the size classes (Figure 5b). Sites not in recovery either had somewhat left‐shifted distributions of size and biomass (Figure 5c,d) or highly irregular size distributions shaped by the site's few remaining individuals (Figure 5e,f). These irregular distributions had many absent size classes, and relative abundances between present size classes were highly variable. Only three sites (Damnation Creek, CA North; Andrew Molera, CA Central; and Government Point, CA South) had size and biomass distributions resembling their respective pre‐SSWS distributions by 2017. Distributions in Southern California and the Channel Islands were nearly all irregular, while all other regions had a mix of sites with left‐shifted or irregular distributions.

**Figure 6:**
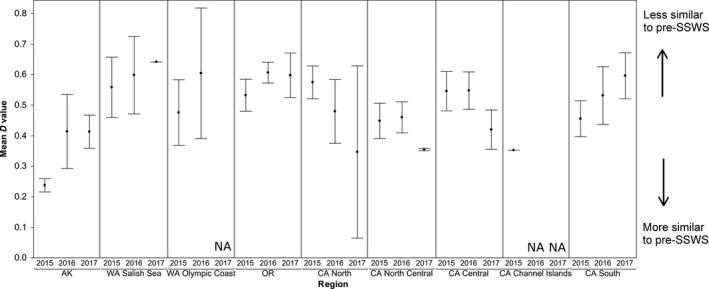
Regional mean similarity of *Pisaster ochraceus* size structure to the long‐term pre‐SSWS (1989–2012) mean size structure. Point heights represent the average *D* statistic of the K–S test (i.e., difference between current and pre‐SSWS distributions) for all sites in a region from 2015 to 2017. Error bars denote standard error. A single line for error bars indicates that only one site in that region had size data that year. The WA Olympic Coast region had no size data in 2017. The CA Channel Islands had no size data in 2016 and 2017

We observed regional differences in directional change in size distribution. From 2015 to 2017, size distributions shifted closer to the pre‐SSWS distribution (represented by decreasing *D*‐values of K–S test) in North California, North Central California, and Central California. Size distributions shifted further away from the pre‐SSWS distribution (represented by increasing *D*‐values) in all other regions (Figure 6). Magnitude of these shifts was not uniform across all sites in a region. In Washington Salish Sea, Oregon, and all California regions, we observed within‐region variability in the direction and magnitude of shifts in size distributions. Washington Olympic Coast and Southeast Alaska experienced only shifts away from the pre‐SSWS distribution, although both regions had substantial variation within them, indicated by large standard errors (Figure 6).

## 4 DISCUSSION

### 4.1 Factors influencing regional trends in recovery

Despite site‐level differences, recovery of *Pisaster ochraceus* populations and their predation potential has begun at regional levels in Washington Salish Sea, Oregon, and for the majority of the California coast north of Point Conception. Individuals produced during and after the outbreak have brought abundances to levels approaching or even exceeding their long‐term averages, but predation pressure in terms of biomass is still at <40% of pre‐SSWS levels at most sites outside of North Central California.

The regional variability in percent of recovering sites does not indicate a single explanatory factor for drivers of sea star recruitment. Because increases in relative count yielded less than 0.67:1 changes in relative biomass (Figure 4), it suggests that abundances alone are a poor metric of ecological interaction strengths of sea stars of different sizes. Even if the populations grow to their pre‐SSWS numbers of individuals, the shift in size structure to domination by smaller sea stars (Figure 5c,d) constitutes a reduction in predation pressure due to lower biomass of juveniles. The regional differences in time elapsed since the mass mortality period influenced time available to detect recovery in these initial postoutbreak years. Mass mortality began in Central California and Washington's Puget Sound area in Fall 2013, while SSWS did not have substantial impacts on the Oregon coast until Summer 2014, at least 9 months later (MARINe 2013; Menge et al., 2016). Assuming the mass mortality period's duration was similar between regions, the time available for postoutbreak reproduction differs by the same amount, potentially giving Central California and the Washington Olympic Coast an earlier start on recovering. In addition, the severity of SSWS mortality varied dramatically between regions. Central and Southern California experienced mortalities of over 95% at many of their sites, while the Oregon coast experienced mortalities closer to 70% (Menge et al., 2016). Washington Salish Sea and Puget Sound experienced a 67% decline in sea star populations, with zero *P. ochraceus* found at some monitoring sites (Eisenlord et al., 2016). These regions have the most information on severity of mortality, while in other regions, less is known about SSWS mortality. The disjunct nature of SSWS appearance combined with the variation in mortality likely contributes to the lack of temporal coherence in spatial patterns of sea star recovery. Our results are also restricted to rocky intertidal sites. Recovery patterns may differ when including sea star populations from a broader suite of sea star habitats, particularly in Alaska and Washington Salish Sea where intertidal habitats are not fully represented by our limited number of sampling sites in these regions (C. M. Miner, personal communication). Additionally, Washington Olympic Coast and the Channel Islands did not have available data in 2017.

Post‐disease regional variations in the magnitude of recruitment may contribute to the geographical variation in the number of sites “in recovery.” At a regional level, North California and Oregon experienced extremely high numbers of sea star recruits in 2015 (Menge et al., 2016). It is likely this high recruitment was in part responsible for the high percentage of recovering sites in these regions (Figure 2b,c). PISCO sea star surveys in spring 2014 showed high numbers of juvenile sea stars (<25 mm) in the Monterey Bay area of Central California, but a relatively small percentage of them were detected in the next largest size class the following spring (Figure S2), suggesting that small individuals did not survive in large numbers until 2015 or later. It is possible that the first wave of recruits (<15 mm) experienced high mortality from residual SSWS, particularly in the warm temperatures of late summer and early fall. Central California's recruit survival in 2014 appears low and unlikely to contribute substantially to population or biomass increases, potentially negating the temporal head start from earlier outbreak onset. Many of the 2014 recruits were spawned during the outbreak period, late summer through winter of 2013. PISCO surveys in spring 2015 showed a substantially lower number of recruits, but numbers in medium size classes began to increase in 2016, suggesting they survived to contribute to predation pressure (Figure S2b). These recruits did not arrive postoutbreak and did not technically provide an indication of recovery, as recovery depends on successful reproduction and recruitment after the disease subsides.

Lack of recruitment combined with high adult mortality likely contributed to the lack of recovery and the extremely low sea star density and biomass in Southern California (Figure 2d,g,h). Marine invertebrates exhibit major breaks in magnitude and timing of recruitment at Point Conception, Cape Mendocino, and Cape Blanco (Broitman et al., 2008), due at least in part to oceanographic patterns. Based on these studies, the geographic break in sea star recruitment could be due to the absence of larval transport originating further north. After previous SSWS outbreaks, sites in Southern California and the Channel Islands took nearly a decade to return to preoutbreak *P. ochraceus* densities. It is possible this area will require a similar amount of time to fully recover after the latest outbreak (Blanchette, Richards, Engle, Broitman, & Gaines, 2005).

The variation in sites in recovery within regions (Figure 2) suggests that local factors exert influence on recovery dynamics. Both adult *P. ochraceus* density and recruitment of juveniles often vary by orders of magnitude between sites separated by only a few kilometers (Hart, 2010; Sewell & Watson, 1993). As more time passes after the outbreak, differences in recovery may become more pronounced within and among regions, making influence of local scale dynamics, severity of mortality, and recruitment better defined.

Recruits rarely come from the site where they were spawned due to the long pelagic larval duration, although biogeographic barriers reduce larval transport between regions (Strathmann, 1978). While we cannot ascertain precisely where recruits originated, their source is likely within the same region. Recruits cross regional biogeographic boundaries in sufficient quantities to maintain gene flow (Frontana‐Uribe et al., 2008), but high recruitment across a gene flow barrier without high recruitment in the adjacent region would be unlikely. These geographic patterns of recovery have not aligned with those of other species of Pacific coast sea stars, suggesting that initial post‐SSWS recruitment patterns have been species‐specific (Montecino‐Latorre et al., 2016).

In another example of disease influencing intertidal community structure, rates of recruitment appear to have a substantial influence on rates of species recovery and subsequent community shifts. Changes in intertidal community structure have been documented after outbreaks of withering syndrome in black abalone *Haliotis cracherodii*. Abalone graze on attached and drifting macroalgae in the lower intertidal and subtidal zones (Scheibling, 1994). Grazing action by adults promotes a covering of bare rock or coralline algae, which helps cue settlement of larval abalone. After withering syndrome caused mass mortalities of black abalone in Southern California, grazing action was reduced and the dominant cover shifted to sessile invertebrates and sea urchins. These substrates are unfavorable for recruitment of abalone and other invertebrates, increasing the barriers to recovery of abalone populations and their grazing pressure (Miner et al., 2006). Extremely low recruitment was considered one of the major factors for limited black abalone recovery and sustained changes in low intertidal communities (Miner et al., 2006; Raimondi, Wilson, Ambrose, Engle, & Minchinton, 2002).

### 4.2 Implications of SSWS recovery dynamics for rocky intertidal communities

If the new juveniles survive to adulthood, the shift in *P. ochraceus* size structure and reduction in predation pressure are only temporary. However, the likelihood of changes to intertidal communities is sensitive to the duration of reduced predation pressure, even if those reductions are temporary (Pfister, Paine, & Wootton, 2016). *P. ochraceus* removal experiments of varying durations have demonstrated that below a certain threshold in mussel size, community change is reversible. Hart's (2010) three‐year removal of sea stars produced moderate downward shifts in *Mytilus californianus* lower boundaries, which were reversed 3 years after sea star predation returned. In contrast, Paine (1966, 1974, and 1976) removed *P. ochraceus* for 5 years. During this time, the mussel bed boundary moved substantially lower and many mussels grew beyond the size at which *P. ochraceus* could consume them. This resulted in longer‐term increases in mussel cover and change in intertidal species composition. Regional‐level and site‐level context influence the strength of *P. ochraceus* predation as well. Geographic variation in the growth rates of both sea stars and mussels also influences the amount of time that community changes remain reversible (Kroeker et al., 2016; Phillips, 2007; Sanford, 2002). Wave exposure, local variation in recruitment of mussel species such as *M. californianus*, and frequency of disturbances that disrupt mussel beds will all shape the long‐term impact of temporary sea star reductions on the community (Menge, Berlow, Blanchette, Navarrete, & Yamada, 1994; Phillips, 2007; Sousa, 1984). It is more difficult to predict changes to predation pressure in communities where mussels do not occupy a significant portion of the rocky substrate, as *P. ochraceus* will eat a diversity of sessile and mobile invertebrate species in the absence of their preferred mussel prey (Feder, 1956; Landenberger, 1968; Mauzey, Birkeland, & Dayton, 1968).

These results provide insight into how populations of a keystone species are recovering following disturbance events such as disease. Postoutbreak sea star recruitment, survival of adults, and local factors controlling prey dynamics shape the level of predation pressure that those populations exert in the early years following a mass mortality. We predict that sites and regions that take longer to complete the recovery process will likely experience greater changes in community composition following the outbreak. Understanding regional and local differences in postmortality demographics is a first step in predicting where communities are likely to change before enough time has elapsed to observe those shifts. These insights could be used to inform site choices in existing monitoring efforts to document intertidal communities throughout the recovery process. As the occurrence of epidemics is expected to rise, emphasis on the geographic patterns and dynamics of recovering host populations will become increasingly important to assessing how diseases shape communities.

## CONFLICT OF INTEREST

The authors have no conflict of interests to declare.

## AUTHOR CONTRIBUTIONS

MMM designed study, assisted with collection of data, performed analysis, and wrote the manuscript. PTR guided study design, guided analysis, and provided substantial feedback.
