# Supplementary material for: Reduction and recovery of keystone predation pressure after disease‐related mass mortality
Source: Ecol Evol. 2018 Mar 23;8(8):3952–64. doi: 10.1002/ece3.3953 (PMC5916292; doi:10.1002/ece3.3953)
Supplement: Supplementary file 1 [file ECE3-8-3952-s001.docx]

# Appendix

*Calculation of growth rates for* Pisaster ochraceus

We identified clear pulses of recruits by visually examining abundances for each *P. ochraceus* size class over time in PISCO/MARINe’s Long Term Monitoring plots. Data are publicly available at <http://www.eeb.ucsc.edu/pacificrockyintertidal/interactive-map/index.html>. We used PISCO’s Interactive Map and Graphing Tool to generate visualizations for the size-date-abundance data that could discern the presence of recruitment “pulses” by the presence of a large number of sea stars in small size classes relative to earlier or later years (Fig. A1). We then tracked recruitment pulse over time as the cohort matured and moved up in size classes. A recruitment pulse was not used if data gaps exceeded two years between measurements or if it was less than two years in duration. We stopped tracking cohorts when they reached the size of 90mm, at which point they were indistinguishable from existing adults.

We estimated the change in size over time to calculate growth rate for each pulse. Dates were converted to years since the start of the pulse observation, in which the first observation was considered year 0. We treated all year-size combinations as x-y pairs. We used linear regression to fit a line to the year-size pairs in each pulse. The slope of the best fit line represented the average growth rate of the pulse (mm yr^-1^)_._ If a site had more than one pulse, we used a mixed regression (R 3.3.2) using points from all pulses, including pulse number as a random effect. This integrated multiple pulses into a single growth rate for each site. We recognize that growth rates fluctuate with food availability (Feder 1970), but juvenile *P. ochraceus* in both lab and field settings show approximately linear trends in growth rates (Sewell & Watson 1993; Pilkerton *et al.* 2016).

If a site did not have distinguishable recruitment pulses to use in growth rate calculation, we assigned the growth rates of the nearest neighboring site with a discernable recruitment pulse. We used the (Euclidean) Near function in ArcGIS 10.5 to determine nearest neighboring site.

**Literature Cited**

1.Feder, H.M. (1970). Growth and predation by the ochre sea star, *Pisaster ochraceus* (Brandt). *Ophelia*, 8, 161–185

2.Pilkerton, A., Apple, J., Kohnert, C., Bohlmann, H., Burnett, N., Bay, P., *et al.* (2016). Investigating Patterns in the Growth Dynamics of Pacific Northwest Sea Stars , *E . troschelii* and *P . ochraceus*. In: *Proceedings of the Pacific Estuarine Resesarch Society, 39th Annual Meeting*. Brackendale, B.C.

3.Sewell, M. & Watson, J. (1993). A “source” for asteroid larvae?: recruitment of *Pisaster ochraceus, Pycnopodia helianthoides and Dermasterias imbricata* in Nootka Sound, British Columbia. *Mar. Biol.*, 117, 387–398

**Table S1.** Extent of each geographic region and number of sites in each region with *Pisaster ochraceus* count and size data from PISCO/MARINe surveys used for count and biomass comparisons. Only sites with count data from the pre-SSWS period (2012 and earlier) and data from 2015 to 2017 were used in analysis. An asterisk, ‘*’, indicates that more sites were sampled for sea stars that year but data is not yet available.

| **Region** | **2015** | **2016** | **2017** | **Extent of region** |
| --- | --- | --- | --- | --- |
| AK | 2 | 2 | 2 | Southeast Alaska panhandle |
| CA Central | 14 | 13 | 9 | Pidgeon Point to Point Conception |
| CA Channel Islands | 4 | 2 | 0 | All Channel Islands (offshore) |
| CA North | 6 | 6 | 2 | CA-OR border to Point Arena |
| CA North Central | 7 | 6 | 7 | Point Arena to Pidgeon Point |
| CA South | 17 | 16 | 15 | Point Conception to US-Mexico border (mainland) |
| OR | 5 | 4 | 3 | WA-OR border to OR-CA border |
| WA Olympic Coast | 5 | 2 | 0 | Washington open coastline (not in straits or Puget Sound) |
| WA Salish Sea | 5 | 5 | 1 | Salish Sea and San Juan Islands |
| **Total** | **68*** | **57*** | **41*** |  |

**Table S2.** Number of sites used in size distribution comparisons for each region. Sample size is lower than sites used for count and biomass comparisons (Table A1) because size distributions could not be constructed for sites with a count of 0 *Pisaster ochraceus*.

| **Region** | **N sites 2015** | **N sites 2016** | **N sites 2017** |
| --- | --- | --- | --- |
| AK | 2 | 2 | 2 |
| CA Central | 11 | 12 | 6 |
| CA Channel Islands | 1 | 0 | 0 |
| CA North | 5 | 6 | 2 |
| CA North Central | 6 | 5 | 2 |
| CA South | 8 | 8 | 7 |
| OR | 5 | 4 | 3 |
| WA Olympic Coast | 5 | 2 | 0 |
| WA Salish Sea | 5 | 4 | 1 |
| **Total** | **48** | **43** | **23** |

**Table S3.** Individual sites sampled in each year. ‘1’ indicates the site was sampled. A blank indicates the site was not sampled. An asterisk, ‘*’, indicates that the site was sampled for sea stars that year but data is not yet available. We excluded sites without sampling in two consecutive years from calculation of 2015-to-2016 or 2016-to-2017 changes in count and biomass in the years without data.

| **Site, by region** | **2015** | **2016** | **2017** |
| --- | --- | --- | --- |
| **AK** |  |  |  |
| Pirates Cove | 1 | 1 | 1 |
| Sage Rock | 1 | 1 | 1 |
| **CA Central** |  |  |  |
| Andrew Molera | 1 | 1 | 1 |
| Boat House | 1 | 1 | * |
| Cayucos | 1 | 1 | * |
| Hazards | 1 | 1 | * |
| Hopkins | 1 | 1 | 1 |
| Mill Creek | 1 | 1 | 1 |
| Occulto | 1 |  |  |
| Piedras Blancas | * | * | 1 |
| Point Lobos | 1 | 1 | 1 |
| Point Pinos |  |  | 1 |
| Point Sierra Nevada | 1 | 1 | * |
| Scott Creek | 1 | 1 | 1 |
| Shell Beach | 1 | 1 | * |
| Stairs | 1 | 1 | * |
| Stillwater | 1 | 1 | 1 |
| Terrace Point | 1 | 1 | 1 |
| **CA Channel Islands** |  |  |  |
| Bird Rock | 1 | 1 | * |
| East Point | 1 | * | * |
| Little Harbor | 1 | 1 | * |
| NW Talcott | 1 | * | * |
| **CA North** |  |  |  |
| Cape Mendocino | 1 | 1 |  |
| Damnation Creek | 1 | 1 | 1 |
| Enderts | 1 | 1 | * |
| False Klamath Cove | 1 | 1 | 1 |
| Kibesillah Hill | 1 | 1 |  |
| Shelter Cove | 1 | 1 |  |
| **CA North Central** |  |  |  |
| Bodega | 1 | 1 | 1 |
| Bolinas Point | 1 | 1 | * |
| Chimney Rock |  |  | 1 |
| Del Mar Landing |  |  | 1 |
| Gerstle Cove |  |  | 1 |
| Point Arena |  |  | 1 |
| Point Bonita | 1 | 1 | * |
| Santa Maria Creek | 1 | 1 | * |
| Sea Ranch | 1 | 1 | 1 |
| Slide Ranch | 1 | 1 | * |
| Stornetta | 1 |  | 1 |
| **CA South** |  |  |  |
| Alegria | 1 | 1 | 1 |
| Arroyo Hondo | 1 | 1 | 1 |
| Cabrillo I | 1 |  | * |
| Cardiff Reef | 1 | 1 | 1 |
| Carpinteria | 1 | 1 | 1 |
| Coal Oil Point | 1 | 1 | 1 |
| Crystal Cove | 1 | 1 | 1 |
| Dana Point | 1 | 1 | 1 |
| Government Point | 1 | 1 | * |
| Mussel Shoals | 1 | 1 | 1 |
| Old Stairs | 1 | 1 | 1 |
| Paradise Cove | 1 | 1 | 1 |
| Point Fermin | 1 | 1 | 1 |
| Scripps Reef | 1 | 1 | 1 |
| Shaws Cove | 1 | 1 | 1 |
| Treasure Island | 1 | 1 | 1 |
| White Point | 1 | 1 | 1 |
| **OR** |  |  |  |
| Bob Creek | 1 | 1 | 1 |
| Burnt Hill | 1 | 1 |  |
| Cape Arago | 1 | 1 | 1 |
| Ecola | 1 | * | * |
| Fogarty Creek | 1 | 1 | 1 |
| **WA Olympic Coast** |  |  |  |
| Kydikabbit Point |  | 1 | * |
| Point Grenville | 1 | 1 | * |
| Point of the Arches | 1 | * | * |
| Sokol Point; Chilean Memorial | 1 | * | * |
| Starfish Point | 1 | * | * |
| Taylor Point | 1 | * | * |
| **WA Salish Sea** |  |  |  |
| Hat Island East | 1 | 1 | * |
| Hat Island West | 1 | 1 | * |
| Post Point | 1 | 1 | 1 |
| Saddlebag North Cove | 1 | 1 | * |
| Saddlebag South East | 1 | 1 | * |

**Table S4**. Reference site growth rates (mm/yr).

| **site** | **latitude** | **longitude** | **region** | **mean growth rate (mm/yr)** | **standard error of growth rate** |
| --- | --- | --- | --- | --- | --- |
| Andrew Molera | 36.281 | -121.863 | CA Central | 4.89 | 1.01 |
| Arroyo Hondo | 34.473 | -120.145 | CA South | 13.57 | 5.049 |
| Bob Creek | 44.245 | -124.114 | OR | 11.81 | 3.035 |
| Bodega | 38.318 | -123.074 | CA North Central | 5.53 | 0.813 |
| Cape Mendocino | 40.341 | -124.363 | CA North | 11.19 | 2.366 |
| Carpenteria | 34.395 | -119.558 | CA South | 18.28 | 5.53 |
| Damnation Creek | 41.65249 | -124.128 | CA North | 13.03 | 2.29 |
| False Klamath | 41.59476 | -124.106 | CA North | 7.46 | 0.793 |
| Fogarty Creek | 44.837 | -124.058 | OR | 6.23 | 1.399 |
| Hopkins | 36.621 | -121.907 | CA Central | 20 | 8.281 |
| Kibesillah Hill | 39.60412 | -123.789 | CA North | 4.05 | 1.494 |
| Mill Creek | 35.98 | -121.49 | CA Central | 8.45 | 4.017 |
| Mussel Shoals | 34.35548 | -119.441 | CA South | 31.39 | 4.401 |
| Occulto | 34.881 | -120.64 | CA Central | 5.02 | 2.937 |
| Point Fermin | 33.707 | -118.286 | CA South | 18.31 | 7.786 |
| Scott Creek | 37.046 | -122.238 | CA Central | 12.34 | 5.482 |
| Shaw Cove | 33.545 | -117.8 | CA South | 19.5 | 6.353 |
| Stairs | 34.731 | -120.615 | CA Central | 11.48 | 5.136 |
| Terrace | 36.949 | -122.065 | CA Central | 15.36 | 3.648 |
| White Point | 33.716 | -118.32 | CA South | 8.66 | 3.736 |

**
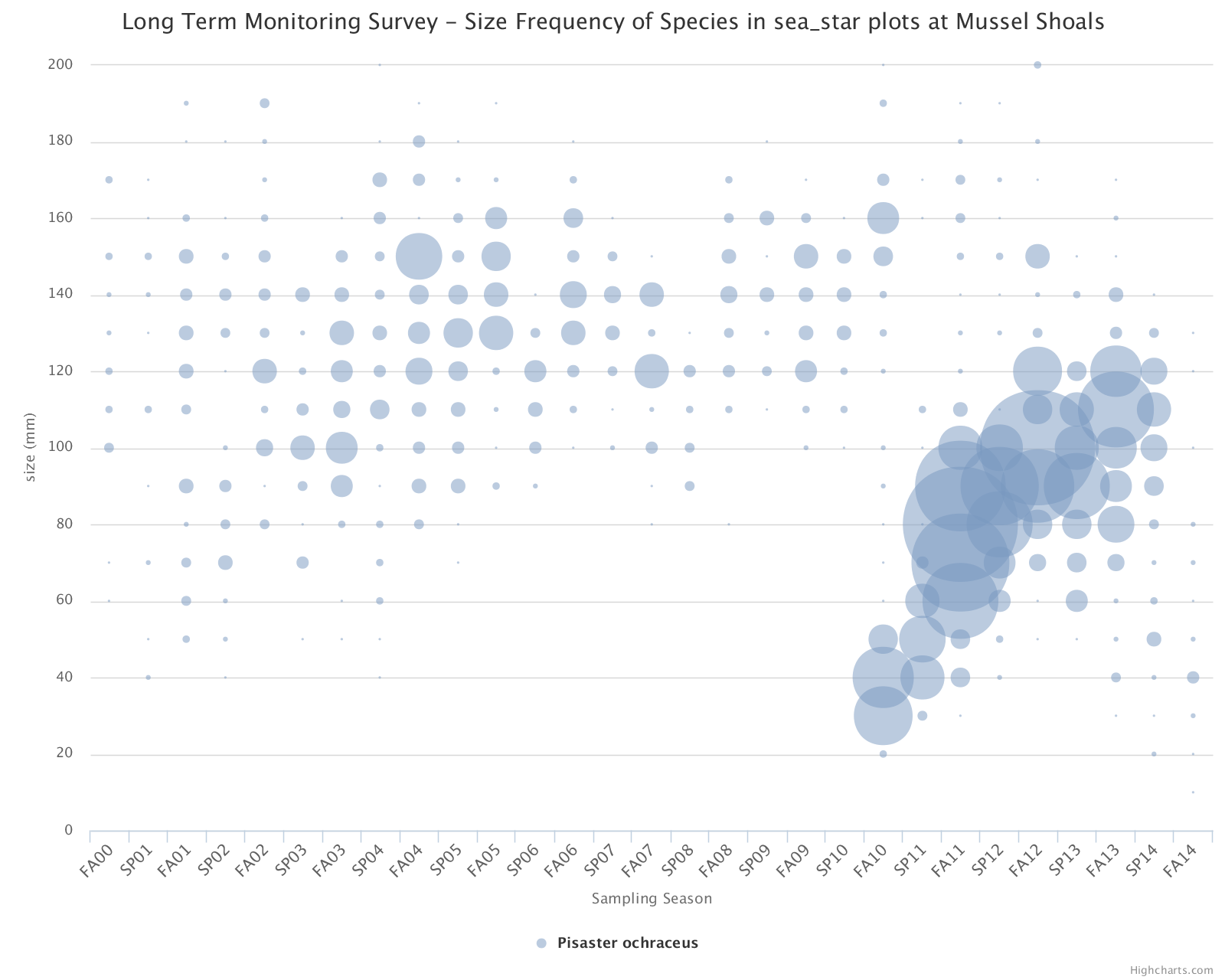
**

**Figure S1.** An example data visualization of *Pisaster ochraceus* abundance in different size classes over time using data from Mussel Shoals, California. Sea star size represents radius of the individual. The diameter of the circle corresponds to the number of sea stars in that size class at the given date. A recruitment pulse is circled in red. Plot courtesy of PISCO’s interactive map and graphing tool at [pacificrockyintertidal.org](http://www.eeb.ucsc.edu/pacificrockyintertidal/interactive-map/index.html).


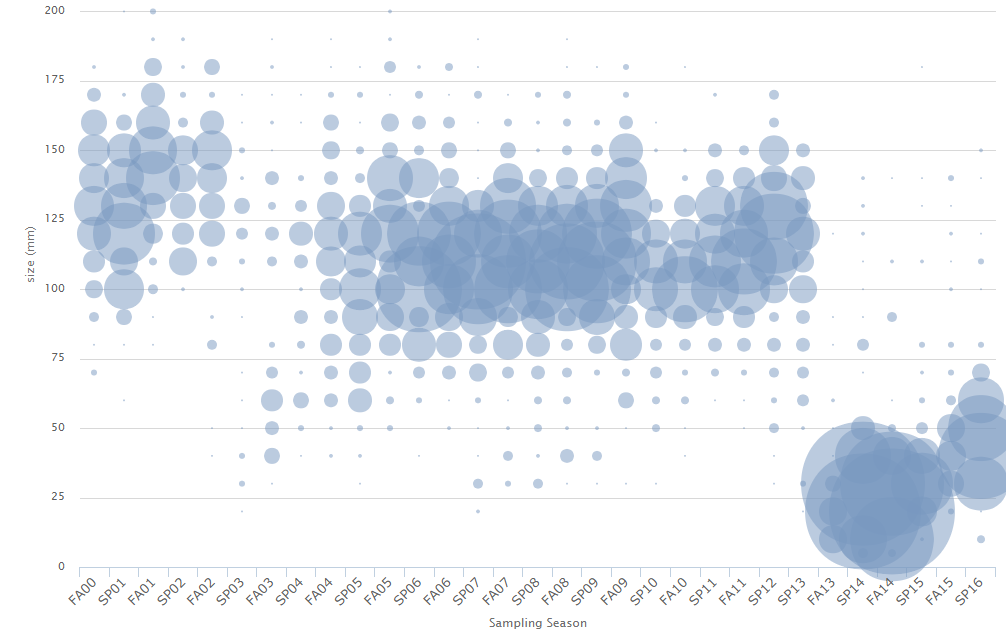


**(a)**

**Figure S2.** (a) Abundance of juvenile size classes of *Pisaster ochraceus* at Terrace Point in Santa Cruz, CA. This site is indicative of overall recruitment trends in the northern Monterey Bay. Sea star size represents radius of the individual. The size of the circles corresponds to the number of individuals in each size class. The area inside the red box is enlarged on the next page. (b) In 2014, a large number of individuals in the smallest size classes appeared, but they did not make it through to the next size classes in substantial numbers in 2015. Plots courtesy of PISCO’s interactive map and graphing tool at [pacificrockyintertidal.org](http://www.eeb.ucsc.edu/pacificrockyintertidal/interactive-map/index.html).


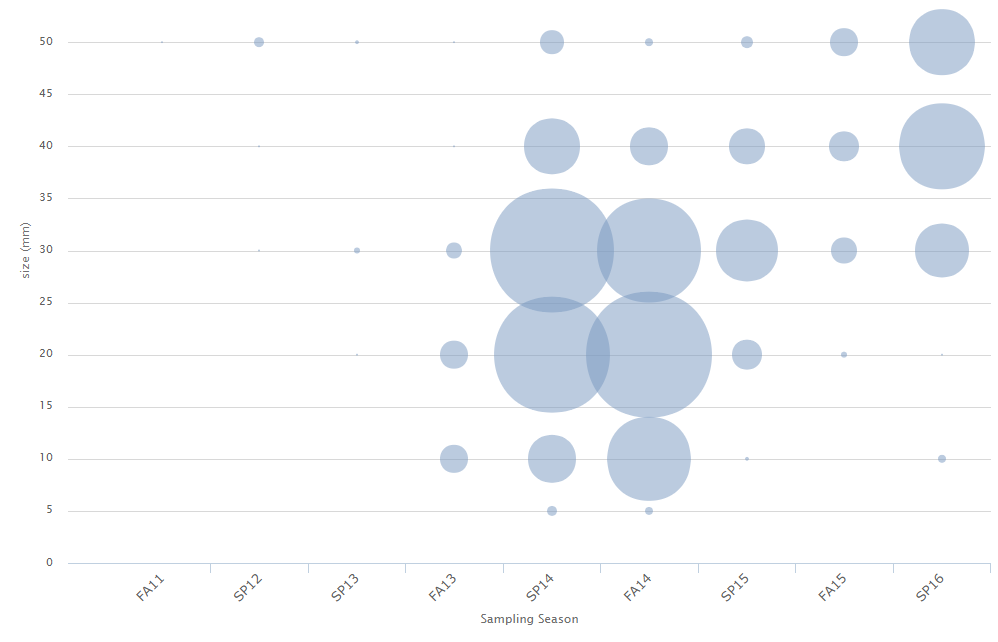


**(b)**

**Figure S3.** Proportion of *P. ochraceus* displaying sea star wasting syndrome symptoms during 2016 site surveys.
